# Malarial Paralysis

**Published:** 1888-08

**Authors:** Arthur W. Hurd

**Affiliations:** Second Assistant Physician to the Buffalo State Asylum for the Insane


### MALARIAL PARALYSIS.*

By ARTHUR W. HURD, A. M., M. D.,

Second Assistant Physician to the Buffalo State Asylum for the Insane.

Various paralyses of supposed malarial origin have been on record for some years, but the cases are so few in number, and some so questionable, that any new matter throwing light on this important subject, is of value. The mere fact that profound paralysis may result from malarial infection, seems fairly well established, but in the matter of differential diagnosis, clinical history, and pathology, much remains to be done. As yet, it appears to be a rare disease, but if it should be learned by closer observation that infantile paralysis is sometimes a malarial manifestation, and curable by anti-periodic remedies, to what importance does this subject then attain? Some works on nervous diseases do not treat of this subject at all, while others give but a few lines. Of recent years, Dr. Gibney, of New York, has given especial attention to this subject, all of his cases being those of children.

The earliest recorded example of intermittent paralysis was made known by Romberg, and I quote his description of it.

"A woman, sixty-four years of age, after being quite well the day before, was suddenly attacked with paralysis of the lower extremities and of the sphincters. Sensibility was unchanged, consciousness clear, the temperature cool, pulse eighty degrees, small and empty; no pain in the spinal cord. The next day there was an astonishing change in the condition, the patient could walk again and void urine voluntarily, and only complained of weakness in the legs. The following morning there was paraplegia again, which had set in at the same hour as it had done two days before. A third paroxysm was awaited, which also set in at the appointed time, although without paralysis of the sphincters. Quinine affected a rapid cure."

There has been under my charge at the Asylum a case whose history warrants a diagnosis of malarial paraplegia; at least, it appears that the foundations for this conclusion are as strong as are those on which unquestioned diagnoses are made constantly.

* Read before the Physicians' Club.

This patient was brought into the office by two men; he could not walk, feet dragged, and he appeared quite helpless. His mental condition was one of dementia. He was easily confused on questioning; could not remember events without great effort, and then imperfectly; complained of pain in the head; inability to concentrate his attention on any subject; showed loss of sensibility in limbs, but no paralysis of bladder or rectum; is twenty-eight years old, and of muscular development.

From his mother, and later from himself, was obtained this history: He was very well up to two and a-half years ago. He then went to live in Rockland county, a malarial district, and became infected. He kept at work for months, however, taking large quantities of quinine. Said that ordinarily his paroxysms were every second day, but that at times they had occurred every seven days, and at one period every fourteen days. Getting no better, he went to Massachusetts in July, 1887, and there suffered severely, being confined to bed. Had paroxysms every second day, and was attended by a physician. One day, during a paroxysm, he suddenly became unconscious, and, it is said, remained in this condition about forty-eight hours. When he regained consciousness, he was found to be paraplegic; he had diminished sensation in limbs; urine and fæces were passed involuntarily. He himself remembers but little of the occurrences at this time. He was taken to the Asylum at Tewksbury, was given quinine again, and got somewhat better, but after a few months, progress being stationary, he was brought home to Hornellsville, thence here in December. He gave no history of syphilis. He was put on anti-malarial treatment, together with tonics, and improvement soon began. In about four weeks he could walk well, with but little uncertainty of gait and without assistance. His mental condition also improved, though more slowly, and his progress has been steady since. He has for several months been on parole, helping about the stable and grounds; shows no trace of paraplegia, and is to-day one of the most active runners in a foot-ball team. Twice since his improvement he has complained of pain in the back (but no paralysis), with general malaise, and each time relief has followed a re-administration of the original remedy.

His mental condition would have permitted discharge, but the pain in the back, which returned, led him to think that he is not entirely over his malarial trouble, and he stays voluntarily until he shall be entirely well.

We have in this case a young man previously healthy, who, two years ago, was attacked with ague, suffered with it some months, and in a paroxysm, without previous nervous or mental symptoms, becomes paraplegic.

In the cases cited by Romberg, Gibney and Ziemssen, the fact that relief followed quinine promptly, is properly given as a strong argument in favor of the malarial origin of these paralyses. If a facial neuralgia disappears promptly under quinine, the average medical mind is convinced that the neuralgia was malarial. Is it because paralysis, one affection of the nervous system, is less common and more unusual than neuralgia, another affection of the nervous system, that the argument which convinces in one, is considered insufficient in the other? This argument cannot be advanced in support of the malarial origin of the paraplegia in the case just cited. He was not given quinine, but decoction of lemon, of the anti-periodic qualities of which much was said in the journals about one year ago, especially in old and obstinate cases of malarial infection. Good results in several instances previously, led to its trial here. Three pints of water, into which one lemon has been sliced, is boiled down to one pint, strained, allowed to stand over night, and given, unsweetened, to the patient before breakfast the following morning.

This was also given the patient on the two occasions when his sensations led him to think he was to have a return of his old malarial trouble, and his experience of so many months made him pretty familiar with them. It is important to note that he had taken quinine in large quantities for a long time, and was completely cinchonized once. At the time of its administration, he said his paraplegia improved to the extent of being able to walk with much difficulty and with dragging feet. The following case is from Dr. Gibney's brochure on this subject:

"Hartwig, also quoted by Erb, relates the case of a vigorous laborer, aged twenty-three, who, five years before, had suffered from a tertian intermittent for a few weeks, but from that time till *November, 1873*, had remained perfectly well and strong. Then he first felt weariness in the legs, this gradually increasing, the arms soon participating. On the third day, he was obliged to take to his bed, and the night following there was complete paralysis of legs, trunk, arms, and even the movements of the head. The muscles of the face escaped, while speaking, breathing and swallowing were somewhat hindered. The sphincters were uninvolved, sensibility was intact, no head symptoms, no pain. Perspiration was excessive. At the end of twenty-four hours, these symptoms subsided, and in half an hour, generally with an increased perspiration, all the muscles again became movable. For the next twenty-four hours he had no sign of paralysis, a weariness and heaviness of limbs being all that he complained of. Then followed an attack of paralysis again, and then there followed regular successive free intervals and attacks of about twenty-four hours' duration. The time occupied by the attack gradually extended to forty hours, the interval being much shorter. Under arsenic, the intervals were lengthened to about forty hours; quinine lengthened them at first to four days, then strychnine, hypodermically, was followed by a re-establishment of the tertian type. A few times the sphincter ani was affected. In one of his attacks, at the end of March, he lay completely paraplegic, only the facial muscles acting normally; the flexors of hands and of feet showed a minimum amount of motion; sensibility of the skin and muscles normal; pulse, seventy-two; respiration, twenty; temperature, ninety-nine and one-half degrees. Reflex action entirely wanting, electrical excitability of the muscles almost entirely extinguished (during the intervals only diminished). Greater and lesser irregularities occurred in the succeeding month; quinine lost its power; yet the disease retained its strongly remittent type, and gradually the cachexia became marked, the muscles showed marked emaciation. The final result of the case is not reported."

There are other cases in adults on record in which intermittent paralysis appeared in the midst of an undoubted malarial attack, and was relieved by quinine, but there is not time to give them at length, and we pass to the manifestation of this trouble in children, and on this point we are indebted to Dr. Gibney, who has collected a few cases. The first case he reports had five attacks of paralysis, and I quote the history of the first two only, and give his summary of the others: Frank P., aged seven years, was brought to the Out-door Department of the Hospital for Ruptured and Crippled, September 28, 1878, for a hydrocele.

"There was observed (at that visit) a peculiarity in gait that excited our interest, though no observations were recorded. Indeed, no history was obtained until some months later, when it was learned that he was one of three children; that phthisis and Bright's disease existed in his malarial parents, and that his mother was of a nervous temperament. I have quite recently learned that for several years she has suffered quite severely at times with various neuralgias. The father is intemperate. Our little patient was living in the summer and early fall at 206 West Thirty-second street, on the first floor of a rear building, and the mother always reported, when asked about hygienic surroundings, that the house was damp, and that malarial fevers prevailed in the immediate neighborhood. In the early part of September he complained of being chilly, and this feeling was followed immediately by heat and vomiting and general indisposition. He had what she termed a "bilious attack." On the following evening he seemed to have high fever, and passed a very restless night, crying aloud repeatedly, and acting like one delirious. There were no convulsions; his bowels were constipated; he referred to the pains to his limbs—in fact, he seemed "sore all over," and cried if he were handled. On getting out of bed next morning he was unable to walk, but could take a few steps after being rubbed. The second night following this he was feverish again, and in the morning he could scarcely use his hands and arms, crying at the same time from muscular pain. During the succeeding week he was confined to bed with a fever; had much cutaneous hyperæsthesia; was at times bordering on delirium; was obstinately constipated, but had no opisthotonos; no symptoms pointing to disturbance of the bladder. He took much quinine at this time. After a week, there was temporary improvement, and he was up for three days, when he fell sick again and was worse, it seemed, than during the first attack. On the subsidence of the fever nearly a week later, paralysis of the four extremities was, to all appearances, complete. Power returned very slowly."

To summarize: His first attack occurred in September, 1878, a few days after an intermittent fever, and that partial recovery took place under the administration of quinine within one week. Indeed, when I first saw the patient for a hydrocele, it was on the twenty-eighth of September, and there was nothing noticeable, except a peculiarity of gait.

The second attack occurred the next month. The recovery was not complete in January, at the time he was admitted to the hospital. Quinine was not employed, and he did not recover until four months after admission. He was simply removed from malarial influences.

The third attack was five weeks after his return home, and he was admitted three days after its invasion, put upon quinine and faradism; recovery being complete this time in three weeks.

The fourth attack, four and one-half months after going home, was preceded one month by the usual "bilious spell," and he did not come into the hospital again until three weeks later, when there was considerable sensory disturbance, requiring morphia for several days.

It was six months this time before he recovered, although quinine was at first used, and then ergot. Be it noted, too, that the recovery was not perfect, inasmuch as the interossei were left over, and have not as yet recovered their tone.

The fifth attack came on six months after discharge from the hospital, coming on gradually, and attended, like the fourth, with pain in the four limbs, and improvement was more marked after he came into the hospital, and now nearly two months have passed, and the cure is not complete.

There are other cases on record whose history goes to support the theory of a direct malarial origin; some coming on during a malarial fever, others following it—some quotidian, others tertian, etc, and often followed by relief under quinine; but space will not allow their enumeration. If this, the malarial, is established as a special form of paralysis, what a gain have we made in treatment!

It may be noted, first, that these attacks appear complicated to say the least, with malarial manifestations; that they come on suddenly; that they are usually bilateral; that muscular electrical reaction is diminished; that recovery from them has been complete.

Are these attacks not malarial, but hysterical? The fact that in hysteria the electrical reactions are normal, while in these the electrical excitability is diminished, together with the fact that there is an absence of convulsions or clinical history of hysteria, would indicate that they are not.

Are they rheumatic? These latter are little likely to assume the form of paraplegia or hemiplegia, and as in the most common form perhaps (Bell's Paralysis), is unilateral, not bilateral.

Can the cases in children be merely unusual cases of polio-myelitis anterior? This, while the most important, is very difficult to decide. The appearance of paralysis after several intermittent and malarial paroxysms; its sometimes intermittent and temporary character; its subsidence, and even recovery, under quinine, are urged as evidences of malarial origin, and not of acute polio-myelitis—for intermittence is not one of the features of infantile paralysis, nor full recovery, unfortunately, one of its results. The intermittence of some cases in adults, the temporary character of the paralysis, and the rapid recovery, are equally against the theory of a myelitis.

Unfortunately, no autopsies have been made, and no definite knowledge of the pathology is obtainable as yet. It is possible that malaria may be one of the causes of anterior polio-myelitis in children, and that the lesions are one and the same. Surely our knowledge of the causes of infantile paralysis is not so accurate that we can exclude this as a possible cause. It has been suggested by Gibney as more fully meeting the clinical history of these cases, that the condition is a blood poison inducing, first, an active hyperæmia of the spinal cord, and, later, a passive condition and possible œdema—a congestion analogous to that of the spleen in this same malarial condition.

The whole subject requires study and observation on the part of the profession, and should it be demonstrated that there *are* paralyses hitherto considered incurable, both in children and adults, which are curable under a different treatment, the labor will not have been without beneficent results.

